# The Exact Distribution of the Condition Number of Complex Random Matrices

**DOI:** 10.1155/2013/729839

**Published:** 2013-11-25

**Authors:** Lin Shi, Taibin Gan, Hong Zhu, Xianming Gu

**Affiliations:** ^1^School of Automation Engineering, University of Electronic Science and Technology of China, Chengdu, Sichuan 611731, China; ^2^School of Mathematical Sciences, University of Electronic Science and Technology of China, Chengdu, Sichuan 611731, China

## Abstract

Let *G*_*m*×*n*_ (*m* ≥ *n*) be a complex random matrix and *W* = *G*_*m*×*n*_^*H*^ *G*_*m*×*n*_ which is the complex Wishart matrix. Let *λ*_1_ > *λ*_2_ > …>*λ*_*n*_ > 0 and *σ*_1_ > *σ*_2_ > …>*σ*_*n*_ > 0 denote the eigenvalues of the *W* and singular values of *G*_*m*×*n*_, respectively. The 2-norm condition number of *G*_*m*×*n*_ is $\kappa_{2}\left(G_{m\times n}\right)=\sqrt{\lambda_{1}/\lambda_{n}}=\sigma_{1}/\sigma_{n}$. In this paper, the exact distribution of the condition number of the complex Wishart matrices is derived. The distribution is expressed in terms of complex zonal polynomials.

## 1. Introduction

 Over the past decade, multiple-input and multiple-output (MIMO) systems have been at the forefront of wireless communications research and development, due to their huge potential for delivering significant capacity compared with conventional systems [1–13]. The capacity and performance of practical MIMO transmission schemes are often dictated by the statistical eigenproperties of the instantaneous channel correlation matrix *W* = *G*_*m*×*n*_^*H*^ *G*_*m*×*n*_, where *G*_*m*×*n*_ is a complex Gaussian matrix and *W* is known to follow a complex Wishart distribution.

In recent years, the statistical properties of Wishart matrices have been extensively studied and applied to a large number of MIMO applications. In statistics, the random eigenvalues are used in hypothesis testing, principal component analysis, canonical correlation analysis, multiple discriminant analysis, and so forth (see [7]). In nuclear physics, random eigenvalues are used to model nuclear energy levels and level spacing [6]. Moreover, the zeros of the Riemann zeta function are modeled using random eigenvalues [6]. Condition numbers arise in theory and applications of random matrices, such as multivariate statistics and quantum physics.

Let *G*_*m*×*n*_ (*m* ≥ *n*) be a complex random matrix whose elements are independent and identically distributed (i.i.d.) standard normal random variables. As we know, the *n* × *n* complex random matrix *W* = *G*_*m*×*n*_^*H*^ *G*_*m*×*n*_ is a complex Wishart matrix. Its distribution is denoted by *W∼W*(*m*, Σ), Σ = *σ* ^2^ *I*. In addition, *W* is a positive definite Hermitian matrix with real eigenvalues; let *λ*_1_ > *λ*_2_ > ⋯>*λ*_*n*_ > 0 and *σ*_1_ > *σ*_2_ > ⋯>*σ*_*n*_ > 0 denote the eigenvalues of the *W* and singular values of *G*_*m*×*n*_, respectively. The 2-norm condition number of *G*_*m*×*n*_ is $\kappa_{2}(G_{m\times n})=\sqrt{\lambda_{1}/\lambda_{n}}=\sigma_{1}/\sigma_{n}$; thus, *κ*_2_(*W*) = *κ*_2_(*G*_*m*×*n*_)^2^.

The exact distributions of the condition number of a 2 × *n* matrix whose elements are independent and identically distributed standard normal real or complex random variables are given in [5] by Edelman. Edelman also obtained the limiting distributions and the limiting expected logarithms of the condition numbers of random rectangular matrices whose elements are independent and identically distributed standard normal random variables. The exact distributions of the condition number of Gaussian matrices are studied in [1] for real random matrix. Here, we derive the exact distribution of the condition number of a complex random matrix for special case Σ = *σ* ^2^ *I*.

This paper is arranged as follows.  Section 2 gives some preliminary results to the complex random matrices. In Section 3, the main result of this work, the density function of *κ*_2_(*W*_*n*×*n*_) for complex case, is proved.

## 2. Some Preliminary Results

 In this section, we give some results on joint density of the eigenvalues of a complex Wishart matrix *W* and *W∼W*(*m*, Σ), Σ = *σ* ^2^ *I*. The determinant, trace, and norm of a square matrix *B* are denoted by |*B*|, tr⁡(*B*), and ||*B*||, respectively.

For any nonnegative integer *p*, a portion of *p* is a multiple (*a*_1_, *a*_2_,…, *a*_*n*_), where *a*_1_ ≥ *a*_2_ ≥ ⋯≥*a*_*n*_ ≥ 0 such that ∑_*i*=1_^*n*^ *a*_*i*_ = *p*, *𝒟*_*p*_ is the set of all portions of *p*, and the symbol ∑_*p*_ *a*_*κ*_ means the summation over *𝒟*_*p*_; that is, ∑_*p*_ *a*_*κ*_ = ∑_*κ*∈*𝒟*_*p*__ *a*_*κ*_.

Let *κ* be any portion of *p*, and let *μ*_1_ > *μ*_2_ > ⋯>*μ*_*n*_ be the eigenvalues of an *n* × *n* matrix *B* as follows:

(1) $$\begin{array}{c} \alpha_{\left[\kappa \right]}=p!\frac{\prod_{i<j}^{n}\left(a_{i}-a_{j}-i+j\right)}{\prod_{i<j}^{n}\left(a_{i}+n-i\right)!},\\ \alpha_{\kappa }\left(B\right)=\frac{\left|\left[\left(\mu_{i}^{a_{j}+n-j}\right)\right]\right|}{\left|\left[\left(\mu_{i}^{n-j}\right)\right]\right|}. \end{array}$$

The zonal polynomials (also called Schur polynomials) of *B* are defined by

(2) $$\begin{array}{c} C_{\kappa }\left(B\right)=\alpha_{\left[\kappa \right]}\cdot \alpha_{\left[\kappa \right]}\left(B\right) \end{array}$$

The following is the basic properties of the zonal polynomials [8]:

(3) $$\begin{array}{c} {\left[\mathrm{tr}\left(B\right)\right]}^{p}=\sum_{p}C_{\kappa }\left(B\right)=\sum_{\kappa \in {𝒟}_{p}}C_{\kappa }\left(B\right). \end{array}$$

The zonal polynomial of the identity matrix is defined by

(4) $$\begin{array}{c} C_{\kappa }\left(I_{n}\right)=2^{2p}p!{\left[\frac{1}{2}n\right]}_{\kappa }\frac{\prod_{i<j}^{r}\left(2a_{i}-2a_{j}-i+j\right)}{\prod_{1}^{r}\left(2a_{i}+r-i\right)!}, \end{array}$$

where

(5) $$\begin{array}{c} {\left[\frac{1}{2}n\right]}_{\kappa }=\prod_{i=1}^{n}{\left(\frac{1}{2}\left(n-i+1\right)\right)}_{a_{i}},\\ {\left(x\right)}_{a_{i}}=x\left(x+1\right)\cdots \left(x+a_{i}-1\right),\;{\left(x\right)}_{0}=1. \end{array}$$

**Definition 1**

For *W* = *G*_*m*×*n*_^*H*^ *G*_*m*×*n*_, where *W* is positive definite Hermitian matrix with real eigenvalues, let *G*_*m*×*n*_ be a complex random matrix whose elements are independent and identically distributed (i.i.d.) standard normal random variables. Let *λ*_1_ > *λ*_2_ > ⋯>*λ*_*n*_ > 0 be the eigenvalues of *W* and Λ = diag⁡(*λ*_1_, *λ*_2_,…, *λ*_*n*_). If *W∼W*(*m*, Σ), Σ = *σ* ^2^ *I* with *m* ≥ *n*, then the joint density of its eigenvalues is defined [2] as follows:

(6) f(Λ)=πn(n−1)(σ2)−mnΓn(n)Γn(m)×∏i=1nλim−n∏i<ln(λi−λl)2exp⁡(−1σ2∑i=1nλi),

where

(7) $$\begin{array}{c} \Gamma_{n}\left(a\right)=\pi^{n(n-1)/2}\prod_{i=1}^{n}\Gamma \left(a+k+1\right),\\ \Gamma \left(x\right)=\int_{0}^{+\infty }t^{x-1}e^{-t}dt. \end{array}$$

**Lemma 2**

For an *n* × *n* matrix *B*, the product of two zonal polynomials can be expressed in terms of a weighted combination of another zonal polynomial [7]; that is, for all *ν* ∈ *𝒟*_*s*_ and ∀*κ* ∈ *𝒟*_*p*_, one has

(8) $$\begin{array}{c} C_{\nu }\left(B\right)C_{\kappa }\left(B\right)=\sum_{\tau \in {𝒟}_{t}}g_{\nu ,\kappa }^{\tau }C_{\tau }\left(B\right), \end{array}$$

where *t* = *s* + *p* and *g*_*ν*,*κ*_^*τ*^ is a constant coefficient.

**Lemma 3**

For an *m* × *n* matrix *B* and for all *κ* ∈ *𝒟*_*p*_, one has [1]

(9) $$\begin{array}{c} {\left|I-B\right|}^{m-n}C_{\kappa }\left(B\right)=\frac{\sum_{s=0}^{\infty }\sum_{\nu \in {𝒟}_{s}}\sum_{\tau \in {𝒟}_{t}}{\left(n-m\right)}_{\nu }g_{\nu ,\kappa }^{\tau }C_{\tau }\left(B\right)}{s!}, \end{array}$$

where *t* = *s* + *p* and *g*_*ν*,*κ*_^*τ*^ is a constant coefficient.

**Lemma 4**

Let *Z* = diag⁡(*ξ*_2_,…, *ξ*_*n*_), *Z*_1_ = diag⁡(1, *ξ*_2_,…, *ξ*_*n*_) and *D* = {1 > *ξ*_2_ > ⋯>*ξ*_*n*_ > 0}, then [2]

(10) ∫D|Z|b−n∏i=2n(1−ξi)2∏i<ln(ξi−ξl)2Cκ(Z1)∏i=2ndξi =(nb+p)Γn(n)πn(n−1)Γn(b,κ)Γn(n)Γn(b+n,κ)Cκ(I),

where

(11) $$\begin{array}{c} \Gamma_{n}\left(a\right)=\pi^{n(n-1)/2}\prod_{i=1}^{n}\Gamma \left(a+k+1\right),\\ \Gamma_{n}\left(b,\kappa \right)=\pi^{n(n-1)/2}\prod_{i=1}^{n}\Gamma \left(a+k_{i}-i+1\right). \end{array}$$

## 3. Main Result

 The exact distribution of the 2-norm condition number of the Wishart matrix *W* is derived by the following.

**Theorem 5**

Let *G*_*m*×*n*_ be a complex random matrix and *W* = *G*_*m*×*n*_^*H*^ *G*_*m*×*n*_. *λ*_1_ and *λ*_*n*_ are the maximum and minimum eigenvalues of *W*, *κ*_2_(*W*) = *λ*_1_/*λ*_*n*_. Then, the exact distribution of *κ*_2_(*W*) is given by

(12) f(κ2(W))=πn(n−1)(σ2)−mnΓn(n)Γn(m)exp⁡(−nσ2λ1)×∑p=0∞∑ κ∈𝒟pλ1mn−n+pσ2pp!∑s=0∞∑ ν∈𝒟s∑ τ∈𝒟t1s!(n−m)ν×gν,κτCτ(κ2(W)−1)(n−1)(n+1)+p+s−1×κ2(W)−(n−1)(n+1)−p−s+1×(n2−1+p+s)×Γn−1(n−1)π(n−1)(n−2)×Γn−1(n+1,τ)Γn−1(n−1)Γn−1(2n,τ)Cτ(I),

where *t* = *s* + *p*.

**Proof**

By making the transformation *λ*_1_ = *λ*_1_, *γ*_*i*_ = 1 − *λ*_*i*_/*λ*_1_, *i* = 2,…, *n*, where 1 > *γ*_*n*_ > ⋯>*γ*_2_ > 0, we obtain the joint distribution of *λ*_1_ and *γ*_2_,…, *γ*_*n*_, as follows:

(13) πn(n−1)(σ2)−mnΓn(n)Γn(m)exp⁡(−nσ2λ1)λ1mn−n|A|2|I−A|m−n ×∏k<ln(γk−γk)2exp⁡[λ1σ2tr⁡(A)].

By using Taylor's formula,

(14) πn(n−1)(σ2)−mnΓn(n)Γn(m)exp⁡(−nσ2λ1)λ1mn−n|A|2|I−A|m−n ×∏k<ln(γk−γk)2∑p=0∞1p![λ1σ2tr⁡(A)]p.

By using property (3), we have

(15) πn(n−1)(σ2)−mnΓn(n)Γn(m)exp⁡(−nσ2λ1)|A|2|I−A|m−n ×∏i<ln(γi−γl)2∑p=0∞∑ κ∈𝒟pλ1mn−n+pCκ(A)σ2pp!,

where *A* = diag⁡(*γ*_2_,…, *γ*_*n*_).

By making the transformation *ξ*_*i*_ = *γ*_*i*_/*γ*_*n*_, *i* = 2,…, *n* − 1, using Lemma 3, and integrating over the set 1 > *ξ*_*n*−1_ > ⋯>*ξ*_2_ > 0, we have

(16) ∫1>γn−1>⋯>γ2>0|A|2|I−A|m−nCκ(A)∏i<ln(γi−γl)2∏i=2n−1dγi =[∑s=0∞∑ ν∈𝒟s∑ τ∈𝒟t1s!(n−m)νgν,κτγn(n−1)(n−2)+p+s−1]  ×∫1>ξn−1>⋯>ξ2>0|Z|2Cτ(Z1)         ×∏i=2n−1(1−ξi)2∏i,j=2n−1(ξi−ξj)2∏i=2n−1dξi,

where *Z* = diag⁡(*ξ*_2_,…, *ξ*_*n*−1_) and *Z*_1_ = diag⁡(1, *Z*).

By using Lemma 4, let *n* be replaced by *n* − 1, let *κ* be replaced by *τ*, and let *b* = *n* + 1.

Then,

(17) ∫1>ξn−1>⋯>ξ2>0|Z|2Cτ(Z1)      ×∏i=2n−1(1−ξi)2∏i,j=2n−1(ξi−ξj)2∏i=2n−1dξi =(n2−1+p+s)Γn−1(n−1)πn(n−1)  ×Γn−1(n+1,τ)Γn−1(n−1)Γn−1(2n,τ)Cτ(I).

It follows that the distribution of (*λ*_1_, *γ*_*n*_) is given by

(18) f(λ1,γn)=πn(n−1)(σ2)−mnΓn(n)Γn(m)exp⁡(−nσ2λ1)×∑p=0∞∑ κ∈𝒟pλ1mn−n+pσ2pp!∑s=0∞∑ ν∈𝒟s∑ τ∈𝒟t1s!(n−m)ν×gν,κτγn(n−1)(n+1)+p+s−1(n2−1+p+s)×Γn−1(n−1)π(n−1)(n−2)Γn−1(n+1,τ)Γn−1(n−1)Γn−1(2n,τ)Cτ(I).

Note that *κ*_2_(*W*) = *λ*_1_/*λ*_*n*_ = (1 − *γ*_*n*_)^−1^; then, the distribution of *κ*_2_(*W*) = (1 − *γ*_*n*_)^−1^ is given by

(19) f(κ2(W))=πn(n−1)(σ2)−mnΓn(n)Γn(m)exp⁡(−nσ2λ1)×∑p=0∞∑ κ∈𝒟pλ1mn−n+pσ2pp!∑ s=0∞∑ ν∈𝒟s∑ τ∈𝒟t1s!(n−m)ν×gν,κτCτ(κ2(W)−1)(n−1)(n+1)+p+s−1×κ2(W)−(n−1)(n+1)−p−s+1×(n2−1+p+s)×Γn−1(n−1)π(n−1)(n−2)×Γn−1(n+1,τ)Γn−1(n−1)Γn−1(2n,τ)Cτ(I).

## 4. Conclusions

 In this paper, the exact distribution of the condition number of complex Wishart matrices is derived. The distribution is expressed in terms of complex zonal polynomials. This distribution plays an important role in numerical analysis and statistical hypothesis testing.

## References

[1] Anderson W, Wells MT (2009). The exact distribution of the condition number of a gaussian matrix. *SIAM Journal on Matrix Analysis and Applications*.

[2] Ratnarajah T, Vaillancourt R, Alvo M (2005). Eigenvalues and condition numbers of complex random matrices. *SIAM Journal on Matrix Analysis and Applications*.

[3] James AT (1960). The distribution of the latent roots of the covariance matrix. *Annals of Mathematical Statistics*.

[4] Chen Z, Dongarra JJ (2005). Condition numbers of Gaussian random matrices. *SIAM Journal on Matrix Analysis and Applications*.

[5] Edelman A (1988). Eigenvalues and condition numbers of random matrices. *SIAM Journal on Matrix Analysis and Applications*.

[6] Mehta ML (1991). *Random Matrices*.

[7] Muirhead RJ (1982). *Aspects of Multivatiate Statistical Theory*.

[8] James AT (1964). Distributions of matrix variate and latent roots derived from normal samples. *Annals of Mathematical Statistics*.

[9] Matthaiou M, McKay MR, Smith PJ, Nossek JA (2010). On the condition number distribution of complex wishart matrices. *IEEE Transactions on Communications*.

[10] Kang M, Alouini MS (2003). Largest eigenvalue of complex wishart matrices and performance analysis of MIMO MRC systems. *IEEE Journal on Selected Areas in Communications*.

[11] Jin S, Mckay MR, Gao X, Collings IB (2008). MIMO multichannel beamforming: SER and outage using new eigenvalue distributions of complex noncentral Wishart matrices. *IEEE Transactions on Communications*.

[12] Zanella A, Chiani M, Win MZ (2009). On the marginal distribution of the eigenvalues of wishart matrices. *IEEE Transactions on Communications*.

[13] Matthaiou M, Laurenson DI, Wang CX (2009). On analytical derivations of the condition number distributions of dual non-central Wishart matrices. *IEEE Transactions on Wireless Communications*.

